# Fertility in four regions spanning large contrasts in serum levels of widespread persistent organochlorines: a cross-sectional study

**DOI:** 10.1186/1476-069X-4-26

**Published:** 2005-11-09

**Authors:** Gunnar Toft, Anna Axmon, Aleksander Giwercman, Ane Marie Thulstrup, Anna Rignell-Hydbom, Henning Sloth Pedersen, Jan K Ludwicki, Valentina Zvyezday, Andery Zinchuk, Marcello Spano, Gian Carlo Manicardi, Eva C Bonefeld-Jørgensen, Lars Hagmar, Jens Peter Bonde, INUENDO

**Affiliations:** 1 Department of Occupational Medicine, Aarhus University Hospital, Noerrebrogade 44, build. 2C, DK – 8000 Aarhus C, Denmark; 2 Department of Occupational and Environmental Medicine, Lund University Hospital, SE – 22185 Lund, Sweden; 3 Scanian Andrology Centre, Fertility Centre, Malmö University Hospital, SE – 205 02 Malmö, Sweden; 4 The Primary Health Care Clinic, P.O. Box 1001, DK-3900 Nuuk, Greenland; 5 Department of Environmental Toxicology, National Institute of Hygiene, Chocimska 24, PL – 00-791 Warsaw, Poland; 6 Problem Laboratory of Human Reproduction, Kharkiv State Medical University, Klochkovskaya Street 156-A, r. 14, 61145 Kharkiv, Ukraine; 7 Section of Toxicology and Biomedical Sciences, ENEA Casaccia, Via Anguillarese 301, I – 00060 Rome, Italy; 8 Laboratory di Genetica, Dip. di Science Agrarie, University of Modena and Reggio Emilia, Viale Kennedy 17, I – 42100 Reggio Emilia, Italy; 9 Unit of Environmental Biotechnology, Department of Environmental and Occupational Medicine, Institute of Public Health, Vennelyst Boulevard 6, Build. 260, University of Aarhus, DK-8000 Aarhus, Denmark

## Abstract

**Background:**

Persistent organochlorine pollutants (POPs) may interfere with reproductive function but direct evidence in humans is very limited.

**Methods:**

Fertility was examined in four regions with contrasting blood levels of POPs. Pregnant women and their partners in Warsaw (Poland), Kharkiv (Ukraine) and Greenland were consecutively enrolled during antenatal visits. Swedish fishermen and their spouses were recruited separately and independently of current pregnancy. Lipid adjusted serum concentrations of 2,2',4,4',5,5'-hexachlorobiphenyl (CB-153) and 1,1-dichloro-2,2-bis (p-chlorophenyl)-ethylene (DDE) were available for both partners. Time to pregnancy interviews were obtained among 2269 women and 798 men provided a semen sample.

**Results:**

Inuits had high levels of both POP markers, Swedish fishermen were high in CB-153 but low in DDE, men from Kharkiv were high in DDE and low in CB-153 while men from Warsaw were low in CB-153 and had intermediate DDE levels. Compared to Warsaw couples, fecundability was reduced among couples from Kharkiv [adjusted fecundability ratio (FR) 0.64 (95% CI 0.5–0.8)] and elevated in Swedish fishermen families [FR 1.26 (95% CI 1.0–1.6)]. Adjusted geometric means of sperm counts and morphology did not differ between regions while sperm motility was higher in men living in Warsaw.

**Conclusion:**

We observed regional differences in time to pregnancy and sperm motility that may be related to regional differences in POP blood levels, but other interpretations are also plausible. In particular, differences in access to safe contraception and in the prevalence of contraceptive failures are most likely to bias comparisons of time to pregnancy.

## Background

In 1993 it was proposed that widespread environmental man-made chemicals with agonistic or antagonistic effects on hormonal steroid receptors, in particular the estrogen receptor, might disturb the foetal development of the male gonads resulting in impaired multiplication of Sertoli cells leading to a permanent reduction of sperm counts [1,2]. Although experimental research offers some support to the hormone hypothesis, the epidemiological evidence on effects of xenobiotics mimicking hormones remains scarce. A recent review of 81 epidemiological studies linking indicators of prenatal serum levels of maternal estrogens with sperm density, hypospadias, cryptorchidism and testicular cancer concluded that – except for testicular cancer – there is no strong epidemiological evidence to indicate that prenatal exposure to estrogens are linked to disturbed development of the male reproductive organs [3,4]. It must be acknowledged, however, that epidemiological studies with adequate exposure assessment of environmental toxicants are very few [3,5].

In this paper we present initial findings from an ongoing, international study that was initiated in order to contribute additional evidence on the environmental hormone hypothesis by studies of persistent organochlorine pollutants (POPs). Polychlorinated biphenyls (PCBs) and the dichlorodiphenyl trichloroethane (DDT) metabolite 1,1-dichloro-2,2-bis (*p*-chlorophenyl)-ethylene (DDE) were selected as exposures of interest because of documented weak hormonal actions [6-15], widespread occurrence worldwide [16], existence of reliable and relatively inexpensive biomarkers suitable for large scale epidemiological studies [17], and the possibility to identify populations and groups with high contrast of exposure [18]. The objective of this paper was to examine whether the regional differences in organochlorine exposure levels are paralleled by differences in couple fertility and semen quality at the population level. In subsequent papers we examine associations between POP concentrations in serum and functional as well as biological indicators of fertility at the individual level [19].

## Materials and methods

### Study design

The basic study design combines four separate interview studies of time to pregnancy with four cross-sectional studies of semen quality using individual measurements of POP serum biomarkers to document exposure gradients between populations.

### Recruitment of study populations

The target populations encompass pregnant women and their male spouses who had antenatal care visits from May 2002 through February 2004 at a large central hospital in Warsaw in Poland, at 3 hospitals and eight antenatal clinics in Kharkiv, Ukraine and at local hospitals in 19 municipalities and settlements throughout Greenland. With few exceptions the antenatal health programs cover all pregnant women in these localities. Altogether 3794 pregnant women and their spouses (if known) from these three localities were informed about the study and encouraged to list up when they, for the first time during the study period, attended the hospitals. Pregnant women were enlisted regardless of earlier reproductive history, parity or gestational age at the time of enrolment. In total 1710 pregnant women (45%) were included in the study (Table 1). The male spouses were consecutively encouraged to collect one semen sample until some 200 men at each site had agreed.

**Table 1:** Target populations and data collection in an international study of fertility.

	**Warsaw**^a^	**Kharkiv**^a^	**Greenland**^a^	**Sweden**^b^	**Total**
**Eligible female target population, n**	**690**	**2478**	**665**	**1439**	**5272**
Interviewed, n	472	640	598	559	2269
Participation rate, %	68	26	90	39	43
Contraceptive failures, %	19	48	6	21	26
Provided valid TTP^c^, n	376	307	520	519	1722
*Stated TTP, n*	*369*	*301*	*455*	*513*	*1638*
*Calculated TTP, n*	*7*	*4*	*57*	*0*	*68*
*TTP in cycles^d^, n*	*0*	*2*	*8*	*6*	*16*
Questionnaire, men^e^, n	472	576	637	195	1880
**Eligible men addressed for the semen study**	**690**	**640**	**256**	**2783**	**4369**
Collected semen samples^f^	198	208	201	191	798
Participation rate, %	29	33	79	7	18
*Sperm motility, manual counting*	*190*	*208*	*200*	*188*	*786*
*Sperm motility, computer assisted*	*165*	*0*	*198*	*179*	*542*
*Sperm morphology*	*197*	*206*	*200*	*184*	*787*

a) Consecutive pregnant woman and their spouses.

b) Retrospective studies of past pregnancies.

c) TTP were considered not valid if the woman was using birth control when becoming pregnant (47 Inuits, 47 Warsaw women, and 297 Kharkiv women), did not provide information on whether she was using birth control (n = 26), was multipara and had not menstruated since her previous pregnancy (n = 16), or had not provided information on whether she had menstruated since her previous pregnancy (n = 5).

d) Possibly censored measure.

e) The total number of individuals with questionnaire information on males. Data were obtained by interviewing the spouse if no male interview was performed.

f) All collected semen samples were analysed for sperm concentration and volume.

In addition to the pregnant couples we included fishermen spouses for a retrospective TTP study addressing the latest planned pregnancy, and fishermen for the cross-sectional semen study. The fishermen and their wives were enrolled independently in two separate steps from a cohort of fishermen living at the west or east coasts of Sweden, which originally was created for purposes of studies of health effects related to high and low level PCB exposure [20,21]. The local ethical committees representing all participating populations approved the study and all subjects signed an informed consent.

### Data collection

At enrolment into the study the women took part in a TTP interview and within one week the men provided a fresh semen sample except for 116 of the men from Greenland who delivered the sample within one year. Both women and men also had a venous blood sample drawn. All interviews as well as sampling, processing, storage and shipment of blood and semen samples were undertaken according to uniform study protocols, questionnaires, and forms. The Swedish questionnaire was slightly different reflecting the population-based nature of this study but the questions establishing the TTP were the same. The data collection in each of the four regions is briefly summarised below.

#### Warsaw, Poland

Pregnant women and their spouses who either visited the obstetric outpatient clinic of the Gynaecological and Obstetric Hospital of the Warsaw School of Medicine or physicians at a collaborating hospital in the same city were informed about the project when attending antenatal classes at the hospitals. The coverage of the hospitals includes central areas of Warsaw as well as suburbs and rural areas close to the city. The antenatal classes typically included some 20–30 women. An obstetrician or midwife informed about the project and encouraged the women to consider participation in the study. Among those 690 women, who, after the classes in an individual talk with the project representative, explicitly accepted or denied the invitation, 472 were enlisted into the study and subsequently took part in a face-to-face TTP interview (participation rate 472/690, 68 %) and 198 of their male spouses agreed to provide a semen sample (participation rate 198/690, 29%). Fifteen midwives performed the TTP interviews when the women were on average 33 weeks pregnant (25–75 percentiles: 31.0–35.7) from September 2002 through March 2004. No data are available on those women and men who did not explicitly accept or decline participation.

#### Kharkiv, Ukraine

Altogether 2478 pregnant women and their spouses who visited one of eight antenatal clinics or three maternity hospitals in Kharkiv, Ukraine, were informed about the project and encouraged to participate. The population covered by the hospitals and clinics are partly living within the city of Kharkiv and partly in adjacent rural areas to the north east of the city. The women were individually contacted by one of 78 gynaecologists from the clinics and hospitals involved in the survey. Among the 2478 couples, 640 women (participation rate 640/2478, 26 %) and 208 of their male spouses (participation rate 208/640, 31%) were enrolled for the TTP and the semen study, respectively, from April 2003 through February 2004. The women were on average 24 weeks pregnant (25–75 percentiles: 12.1–33.6 weeks) weeks when the interview was performed. One of the reasons that a large number of pregnant women refused to participate was concern that the collection of a blood sample (altogether 35 ml whole blood) would imply a risk for the pregnancy and the baby – in particular when anaemia had been detected during pregnancy. Demographic and reproductive information was obtained from a sample of 605 of those women that declined participation in the study. The average age was slightly lower among non-participating women [22.8 (SD 2.4) versus 24.9 (SD 2.8) years], while the average number of children in the two groups was similar (1.1 versus 1.2 among those with at least one child).

#### Greenland

In total, 901 pregnant women from 15 municipalities (Aasiaat, Ilulissat, Kangaatsiaq, Maniitsoq, Nanortalik, Narsaq, Nuuk, Paamiut, Qaanaaq, Qaqortoq, Qasigiannguit, Qeqertarsuaq, Sisimiut, Uummannaq, Tasiilaq), including 4 settlements (Kulusuk, Kuummiut, Saattut and Ukkusissat) representing all regions of Greenland, were listed by the local midwife when visiting the local hospital or health clinic in June 2002 through May 2004. Two hundred and thirty six did not fulfil one or more eligibility criteria (108 women and 140 men were not born in Greenland and 43 were less than 18 years of age). One physician speaking the native language approached the remaining 665 and 598 (participation rate 598/665, 90%) provided a face-to-face TTP interview. The 67 eligible women that were not included in the study were out of range (32 women) or refused to participate (35 women). The age and parity distribution in participating and not participating eligible women were almost identical (data not shown). The women were on average 24 weeks pregnant (25–75 percentiles: 16.7–32.4 weeks) when interviewed. For the semen study 256 male partners were asked to participate. Thirty-five persons did not want to participate and 20 dropped out (18 could not be reached and 2 did not show up after 2 reminders) giving a total of 201semen samples (participation rate 201/256, 79%).

#### Sweden

Altogether 1439 fishermen wives, born 1945 or later, were contacted and 559 (participation rate 559/1439, 39 %) were enrolled for the TTP-study between December 2002 and March 2004. The age distribution among the participants (median 50 years, range 29–64) did not differ from that of those who declined participation (n = 691; median 51 years, range 27–58), or from non-respondents (n = 178; median 49 years, range 32–58). The men in the semen studies were recruited independently. In total 2783 men were asked in writing about their interest in taking part in a semen study and from 191 of these a semen and blood sample was collected during a visit to the residence in a mobile laboratory (participation rate 191/2783, 7%). To allow for comparisons with men recruited when their spouse were pregnant, we only included men that had ever fathered a child (79%).

The recruitment settings are not expected to affect the characteristics of the four study populations as such. For instance, none of the clinics or hospitals differentially received women with high-risk pregnancies. However, the use of an earlier cohort for the Swedish sample resulted in higher age of the fishermen and higher parity of the latest planned TTP study.

### Interviews of female and male participants

We gathered information on TTP for the current (pregnancy based cohorts) or the latest planned pregnancy (the Swedish population based cohort) by face-to-face interviews with the women at the hospital or the residence or by telephone (Sweden). We used a structured interview questionnaire that was developed and validated in an earlier European Concerted Action [22]. After identifying those women that became pregnant without using birth control, TTP was assessed in three different ways. Firstly, the women were asked about their TTP using the following set of questions:

*Leading up to this pregnancy, when was it that you started having sexual intercourse without using any birth control to prevent pregnancy?" Month:______ Year:______. We now call this the "STARTING TIME*".

How long was it from that "STARTING TIME" until you became pregnant? (The date you became pregnant is the date you conceived) How long? Weeks:______ and/or Months:______ and/or Years:______

Of the women who provided information on TTP, 95% supplied information according to this method (Table 1). The questionnaire also contained a question on the date (month and year) when the couple stopped using birth control. Moreover, the date of the last menstrual period was established for all Swedish women as well as for all women from Greenland, Warsaw and Kharkiv who did not use any birth control, or who had menstruated since a previous pregnancy. If the woman had not provided a TTP according to the questions above, these two dates were used to calculate a TTP (4% of all TTPs; Table 1). If information about the day of the month was missing it was defined as the 15 th in the respective month. Finally, the women were asked about how many times they menstruated in the time period when they tried to become pregnant (0, 1, 2, 3 or more than 3 times). If no other measure of TTP was available, this possibly censored measure was used (1% of all TTPs; Table 1).

In addition the interview included questions on demographic and social factors, diet, lifestyle, medical history, job title, and occupational exposures. Information about time varying exposures as tobacco consumption and intake of alcoholic beverages was given with reference to the date when the couple started trying to become pregnant.

Similarly, all male partners that agreed to participate were interviewed to obtain information on lifestyle factors, urogenital disorders, occupational factors as well as questions about periods of abstinence and other issues relating to delivery of a semen sample. These data were used to describe reproductive characteristics of couples with men providing and not providing semen samples. The Swedish subsample was not included in these analyses because of limited overlap between female and male participants and the delayed sampling of semen relative to the latest planned pregnancy.

Both female and male questionnaires were translated to native language in the participating countries and back translated to English for correction of errors that occurred during the translation process. To minimize errors during typing in, all questionnaires were centrally typed in with inconsistencies in 1.7 % of the two sets of typing. When inconsistencies between the two sets of typing occurred the original data was looked up and the typed in data was corrected if necessary.

### Measures and levels of exposure markers

Serum concentrations of 2,2',4,4',5,5'-hexachlorobiphenyl (CB-153) as a biomarker of PCB exposure and DDE as a marker of DDT exposure among 1445 women and 1172 male spouses or fishermen were analysed by gas chromatography mass spectrometry following solid phase extraction. Collection of samples and laboratory methods have been described in detail elsewhere [21,23]. CB-153 and DDE levels were adjusted for serum concentrations of triglycerides and cholesterol that were determined by enzymatic methods [18].

A comprehensive description of exposure profiles is given in [18]. In short, the median lipid adjusted serum concentrations of CB-153 were the highest among Inuit men (200 ng/g lipid), slightly lower in Swedish fishermen (190 ng/g lipid) and substantially lower among men from Kharkiv (44 ng/g lipid) and Warsaw (17 ng/g lipid). On the contrary, the median serum concentrations of p,p'-DDE was highest in Kharkiv (930 ng/g lipid) and lowest in Swedish fishermen (240 ng/g lipid) with Inuit and Polish men falling in between (560 and 530 ng/g lipid). Thus the Inuit men have rather high levels of both PCB and p,p'-DDE, the Swedish fishermen high PCB but low p,p'-DDE, and men from Kharkiv and Warsaw low PCB but rather high p,p'-DDE. Serum levels among women were in general lower but with a regional distribution that paralleled the distribution among the men (data not shown).

### Collection and analysis of semen samples

Semen samples were collected by masturbation at the residence or in privacy in a room at the hospital. The subjects were asked to abstain from sexual activities for at least two days before collecting the sample, and to note the actual abstinence time. If collected at home, the sample was kept close to the body to maintain a temperature close to 37°C when transporting it to the laboratory immediately after collection. The samples were analysed for motility and concentration according to a manual for the project based on the WHO manual for basic semen analysis [24]. All samples were analysed by one researcher in each country and all semen analysers had been trained in a series of three workshops held before and during the sample collection at the Fertility Centre, Malmö University Hospital, Sweden. The variation among analysers was minimal [25] with a median inter-individual CV of 8.1% and 11.1%, respectively, for concentration and motility (grade a+b) assessments. All analyses of sperm motility were initiated within 95 minutes and 95 % of the analyses were initiated within 60 minutes after ejaculation. The morphology of the sperms from all the countries in this project were determined centrally by two technicians at the Fertility Centre, Malmö University Hospital, on Papanicolaou stained smears using the WHO 1999 criteria.

### Statistical analyses

Assuming an average menstrual cycle length of one month, we estimated the probability of clinical pregnancy in a cycle among women not pregnant in the preceding cycle, conditionally that they did become pregnant. Since TTP was measured in discrete times (0, 1, 2, 3 n months), Fecundability Ratios (FR) and the 95% confidence intervals (95% CI) were calculated by Cox proportional hazard regression with handling of ties. The Fecundability Ratio (FR) estimates the fecundability in each region compared to the Warsaw region, which was chosen as reference because it was the region with the lowest POP exposure.

In order to evaluate possible fertility related selection bias we also compared TTP among men providing and not providing semen samples in the three pregnancy based populations where the semen studies were performed among subsets of all couples enrolled into the study (Warsaw, Kharkiv and Inuits). In all analyses couples that became pregnant in spite of contraception or with otherwise undefined or missing TTP values (n= 537) were excluded. TTP values above 12 months (n = 253) were censored to account for medical treatment for infertility, which is usually not started until after one year of infertility. We also performed sensitivity analyses that (i) used censoring after 18 and 24 months, (ii) included couples that became pregnant in spite of use of contraception (TTP assigned 0), (iii) included first parity pregnancies only, (iv) and that included couples than discontinued non-oral contraception only. All analyses were adjusted for established determinants of fecundability, namely female age and female smoking – while parity, other female lifestyle factors, current employment, reproductive disease history, frequency of intercourse and use of oral contraceptive were included in the models if the risk estimate changed more than 10% in any population.

The distribution of semen characteristics in the four populations was compared overall and in pairs by analysis of variance (ANOVA) and multiple linear regression. Sperm concentration, total sperm count, percentages of sperms with normal morphology, age and abstinence time were transformed by the natural logarithmic function, which improved normality and homogeneity of variance. The percentage of motile sperms was not transformed. The mean values for sperm concentration and total sperm count on the logarithmic scale in each region were adjusted for duration of sexual abstinence before delivery of the semen sample in all analyses (PROC GLM, the LSMEANS options). The model fit was evaluated by visual inspection of residual plots. Geometric mean values and their confidence limits were obtained by back transformation. Other potential confounding determinants of semen characteristics as age, spillage, body mass index, fever last three months, season of sampling, urogenital infections or surgery were included in the models if the regression coefficients were changed by more than 10% for any population [26]. Motility analysis was restricted to the 95 % of samples for which the analysis was initiated within 60 minutes after collection.

All analyses were performed using SAS 9.13 software [27]. The term statistically significant is used to denote a p-value less than 0.05.

## Results

Data related to creation of cohorts and participation at various levels are displayed in Table 1. The highest rate of participation in TTP interviews as well as in the semen studies were encountered in Greenland while participation was substantially lower in the other study groups.

Table 2 and 3 presents major differences between the regions with respect to demographic characteristics, lifestyle factors, reproductive behaviour and prevalence of urogenital infections. Thus use and type of contraception was strongly divergent in the four regions. In the Kharkiv cohort, TTP was not defined in almost half of the couples because pregnancies were not planned and occurred in spite of contraception, mostly less safe contraceptive methods (Table 3). However, within regions male and female characteristics were much alike among those couples where the man provided a semen sample compared to those that did not deliver a sample (data not shown).

**Table 2:** Characteristics of women providing time to pregnancy interviews (n = 2269) and men providing semen samples (n = 798).

	**Warsaw**		**Kharkiv**		**Greenland**		**Sweden**	
	Women N = 472	Men N = 198	Women N = 640	Men N = 208	Women N = 598	Men N = 201	Women N = 559	Men N = 191

Age, mean (SD)	29 (4)	30 (4)	25 (5)	26 (5)	27 (6)	31 (7)	29 (5)	47 (10)
Employed, %	89	97	61	75	51	83	70	95
Body mass index, kg/m^2^, mean (SD)	21 (3)	26 (3)	22 (3)	24 (3)	25 (4)	26 (5)	25 (4)	27 (3)
Current smoking, %	19	27	22	66	73	72	31	23
Alcoholic beverage consumption, drinks/ week, mean (SD)	2 (3)	6 (6)	1 (8)	3 (2)	2 (6)	9 (14)	1 (3)	n.a
Coffee, cups/week, mean (SD)	1 (1)	2 (2)	2 (1)	2 (2)	2 (3)	5 (5)	3 (3)	n.a
Urogenital infections^a^, %	14	5	13	5	85	83	27	21
Urogenital disorders^b^, %	21	3	12	3	7	1	44	2
Primi parity, %	91	-	79	-	31	-	4	-
Intercourse daily, %	18	-	19	-	42	-	4	-

a) Chlamydia infection, gonorrhoea, other sexually transmitted diseases or for women: Pelvic infections after delivery; for men: epididymitis or mumps in adulthood.

b) For women: Ovarian cysts, fibroids, myomas, endometriosis, cancer therapy and surgery on uterus, salpinges or ovaries. For men: Treatment for retracted testis, varicocele, testicular torsion, and testicular cancer.

**Table 3:** Type of contraception by categories of time to pregnancy (TTP). Row percentages.

	**The pill**	**Coil or implant**	**Condom or diaphragm**	**Withdrawal, safe periods, other**	**No contra-ception**	**Missing data**
**TTP defined^a^**						
Warsaw n = 376	25	1	31	28	7	8
Kharkiv n = 307	3	1	16	40	16	24
Greenland n = 520	39	16	5	4	27	9
Sweden n = 519	25	29	25	9	0	10
**Contraceptive failures^b^**						
Warsaw n = 89	6	2	41	52	0	0
Kharkiv n = 310	5	3	35	56	0	1
Greenland n = 38	40	8	29	24	0	0
Sweden^c ^n = 150^d^	29	10	15	3	0	42
**Missing TTP data^e^**						
Warsaw n = 7	0	0	43	14	29	14
Kharkiv n = 23	8	4	4	39	4	1
Greenland n = 40	25	5	5	5	18	43
Sweden n = 40	13	3	13	3	0	70

a) Time from a well defined start of unprotected sexual intercourse until last menstrual period

b) Became pregnant in spite of use of contraception

c) Frequencies based upon first unplanned pregnancies

d) The number of unplanned latest pregnancies

e) Including women that became pregnant following delivery without recurrence of menstrual bleeding (n = 16)

The fecundability among couples in Kharkiv was lower than among couples in Greenland and Warsaw and among fishermen families (Table 4). This difference became even more pronounced when the risk estimates were adjusted for female age, female smoking and several other known or suspected risk factors. Fishermen families exhibited increased fecundability although adjustment for potential confounders weakened the association to borderline significance. Similar findings were obtained when TTP values were censored after 18 and 24 months rather than after 12 months, in analyses only including couples not using oral contraception and in analyses only including the first pregnancies except that the latter analysis revealed a reduced fecundability among fishermen's families (Table 4, the foot note). Inclusion of couples that became pregnant in spite of contraception by assigning these couples a TTP value of 0 resulted in weaker differences between regions.

**Table 4:** Fecundability ratios^a ^(FR) for couples in Kharkiv, Greenland and Sweden in comparison with couples in Warsaw.

	**Number pregnant (row percentage)**				**FR crude**	**FR adjusted^b^**	**95% CI**
	**0 – 3 months**	**4 – 6 months**	**6 – 12 months**	**> 12 months**			

**Warsaw**	208 (55)	38 (10)	60 (16)	70 (19)	1.00	1.00	-
**Kharkiv**	147 (48)	37 (12)	39 (13)	84 (27)	0.80	0.64	0.53 – 0.79
**Greenland**	295 (57)	61 (12)	85 (46)	80 (15)	1.07	1.00	0.80 – 1.27
**Sweden**	355 (68)	75 (15)	36 (7)	53 (10)	1.35	1.26	1.01 – 1.57

a) Adjustment for female age (continuous), female current smoking (yes/no), primiparity (yes/no), daily sexual intercourse (yes/no) and current employment (yes/no). All covariates except current employment contributed significantly to the model. Female urogenital disease or infection, low and high body mass index and use of oral contraceptives did not change at least one of the risk estimates with at least 10%.

b) The corresponding age and smoking adjusted FR among nulliparous women were (i) Kharkiv: 0.59 (95% CI, N = 503) (ii) Greenland: 0.90 (95% CI 0.68–1.19, N = 187) and Sweden FR 0.90, (95% CI 0.73 – 1.11), N = 429]. The Swedish risk estimate based upon the first planned pregnancy.

Ranking of regions according to median serum levels of DDE and couple fecundability resulted in identical successions while couple fertility at the regional level seemed unrelated to the average CB-153 levels.

The fecundability decreased with increasing age, was reduced among female smokers, in primi-parae, in less sexually active couples and in women reporting urogenital disorders. Fecundability was slightly lower among couples providing semen samples compared to those not providing samples in all regions, but the difference was not statistically significant in any of the three regions or overall (Table 5).

**Table 5:** Fecundability ratios^a ^(FR) for couples providing semen samples in comparison with couples not providing semen^b^.

	**Number pregnant (row percentage)**				**FR crude**	**FR adjusted**	**95% CI**
	**0 – 3 months**	**4 – 6 months**	**6 – 12 months**	**> 12 months**			

Warsaw							
- sample	123 (56)	23 (10)	37 (17)	38 (17)	1.00	1.00	-
+sample	85 (55)	15 (10)	23 (15)	32 (20)	0.91	0.90	0.7 – 1.1
Kharkiv							
- sample	103 (49)	27 (13)	27 (13)	53 (25)	1.00	1.00	-
+sample	44 (46)	10 (10)	12 (12)	31 (32)	0.87	1.02	0.8 – 1.4
Greenland							
- sample	205 (60)	33 (10)	50 (15)	53 (16)	1.00	1.00	-
+sample	89 (50)	28 (15)	35 (20)	27 (15)	0.89	0.88	0.7 – 1.1
All							
- sample	431 (56)	83 (11)	114 (15)	144 (19)	1.00	1.00	-
+sample	218 (51)	53 (12)	70 (16)	90 (21)	0.89	0.91	0.80 – 1.04

a) Adjustment for region (only the overall estimate), female age (continuous) and current female smoking (yes/no).

b) Sweden not included (TTP and semen studies with marginal overlap).

The crude sperm concentration, the total sperm count, other semen characteristics as well as potential confounding factors recorded at the sampling time are presented in Table 6 for each study population and Table 7 reports crude and abstinence time adjusted geometric mean values with their 95% confidence limits as well as pair-wise comparisons between each region and the reference region (Warsaw).

**Table 6:** Unadjusted semen characteristics by region.

	**Warsaw** ** N = 198**	**Kharkiv** ** N = 208**	**Greenland** ** N = 201**	**Sweden** ** N = 191**
Sperm concentration × 10^6^/ml				
mean (SD)	88 (80)	75 (61)	72 (61)	57 (44)
median (p5 – p95)	64 (7–258)	59 (10–193)	53 (11–178)	49 (9–166)
Total sperm count × 10^6^				
mean (SD)	343 (374)	255 (238)	245 (241)	182 (159)
median (p5 – p95)	197 (19–1071)	181 (24–746)	186 (32–667)	133 (15–500)
Volume ml, mean (SD)	3.8 (1.8)	3.5 (1.9)	3.5 (1.7)	3.3 (1.7)
A+B motile, % (SD)	60 (20)	54 (22)	55 (19)	57 (21)
CASA, motile, % (SD)	42 (22)	not performed	50 (21)	32 (21)
Normal morphology, % (SD)	6.7 (3.8)	7.3 (4.2)	6.9 (3.7)	8.2 (4.5)
Abstinence period ^a^, days,				
mean (SD)	7.7 (9.5)	3.9 (2.0)	3.5 (3.1)	3.7 (2.6)
median (p5 – p95)	4.0 (1.0–30.0)	3.0 (1.5–7.0)	3.0 (0.5–7.0)	3.0 (1.0–8.0)
Time to analysis, minutes, mean (SD)	52 (8)	37 (12)	35 (18)	44 (12)
Season for sperm collection, %				
Spring	19	26	32	34
Summer	10	22	54	35
Fall	21	24	14	31
Winter	50	28	0	0
Fever last three months, %	9	10	13	3
Pain at urination last three months, %	2	19	3	0
Infections, vaccines or surgery last six months, %	26	72	12	8
Medicine or natural medicine use last six months, %	33	68	32	33
Spillage of semen, %	6	17	11	16

a) Abstinence time only included from subjects reporting abstinence time <60 days.

**Table 7:** Geometric mean values and the 95% confidence interval for semen characteristics by region.

	**Warsaw**	**Kharkiv**	**Greenland**	**Sweden**^a^	**P overall, crude/ adjusted^b^**
Sperm concentration, mill/ml	51 (44;58)	55 (49;61)	57 (50;64)	45 (39;52)	0.04/0.08
Urogenital infections	23 (14; 40)	50 (30;82)	**53 (47; 60)**	44 (33;58)	0.22/0.02
No urogenital infection	54 (47;62)	56 (50;64)	62 (46; 83)	47 (40;54)	0.07/0.19
Sperm count^c^, millions	164 (142;188)	179 (155;206)	180 (156;207)	146 (123;173)	0.08/0.22
Motile spermatozoa^d^, %	60 (57; 63)	**54 (52; 57)**	**55 (52; 58)**	56 (53; 60)	0.02/0.02
Normal morphology, %	6.7 (6.2;7.3)	7.1 (6.5;7.6)	7.1 (6.5;7.6)	7.9 (7.2;8.7)	0.07/0.07

Figures in bold indicate p < 0.05 in pair-wise comparisons with the Warsaw sample as reference (Dunnett method).

a) Men that have ever fathered a child.

b) Period of abstinence, count values only.|

c) Restricted to men reporting no spillage.

d) Arithmetical mean values.

The lowest adjusted sperm concentration was found in the Swedish fishermen and the adjusted sperm concentrations differed only slightly among men from Warsaw, Kharkiv and Greenland.

Potential confounders related to sampling and processing of the specimens (season, recent fewer and spillage) changed the effect on regression coefficients of sperm concentration less than 10% in all study groups and was therefore not included in the final model. Among other possible confounders only urogenital infections changed the estimate more than 10%, but only in Greenland, where urogenital infections are much more prevalent (83%) than in the other regions (Swedish fishermen 21% and Warsaw and Kharkiv 5%).

The sperm concentration, stratified by urogenital diseases is presented in Table 7, indicating a reduction in sperm concentration among men that reported urogenital diseases in all of the four study populations with a weighted average reduction of 17.5%. However, only in the samples from Warsaw the difference reached statistical significance. The very low sperm count among men from Warsaw with urogenital infections should be interpreted with caution since this number is based on only 9 subjects.

The distribution of total sperm count (concentration multiplied by volume) within and between regions paralleled sperm concentration. We did not observe obvious differences between regions of crude values (p = 0.08), sperm counts adjusted for trivial factors as period of abstinence (p = 0.22) or sperm counts adjusted for urogenital infections (p = 0.13).

The sperm motility (manual counting) differed among countries (p = 0.02), with the highest motility observed in semen samples from Warsaw and the lowest on semen samples from Kharkiv. The adjusted geometric mean percentage of men with morphological normal sperm differed up to 15% among regions with lowest values in Warsaw and highest values in Sweden (Table 7). Both for motility and percentage of normal sperms none of the tested potential confounders or explanatory covariates changed the estimate more than 10%.

## Discussion

Comparison of health outcomes in regions with highly contrasting exposure levels may provide clues pointing to environmental causes of disease otherwise impossible to detect within populations [18,28]. This international study of fertility includes populations with body burdens of POPs ranking highest in the world and POP contrasts between regions reaching more than one order of magnitude. We observed regional differences in couple fecundability in terms of higher fecundability among Swedish fishermen's families that are characterised by a high PCB but low DDE blood levels and low fecundability in the Kharkiv region characterised by low PCB but high DDE levels. Thus fecundability was related to the average population blood levels of DDE but not PCB. On the contrary, semen quality was remarkable stable across regions except that sperm motility was increased in the Warsaw region having low PCB levels and medium DDE levels.

From these data it is tempting to speculate that persistent environmental pollutants might contribute to the regional variation of fertility. Reproductive function is regulated by hormonal signalling. Several in-vitro and ex-vivo studies indicate that POPs weakly interact with steroid receptors [29-32] and animal studies have reported effects on reproductive organs following administration of both various PCB congeners and mixtures [33,34] and DDT metabolites [35]. Few US and Swedish studies on POP related effects on TTP have been conflicting – some reporting effects and others not [3], but one study addressing partners of male DDT applicators found impaired fecundity [36]. Other compounds or mixtures of environmental toxicants, as for instance phthalates, might be of significance too. CB-153 and DDE are hardly reflecting the total exposure to hormonal active xenobiotics. Thus men living in Warsaw have relatively low levels of CB-153 and DDE but could have higher exposure to other compounds, which have not been considered in the present study. In a recent paper, Hauser et al claims a synergistic effect between PCB and phthalates on semen quality [37], although this finding was not corroborated in a Swedish study of military conscripts [38]. However, at present we have no data to indicate that people in Warsaw are more or less exposed to the wide range of xenobiotics that potentially could interfere with.

Inuits had high-level exposure to both POP markers but yet the fecundability and semen quality was not obviously reduced. It has previously been suggested that the putative negative effect of consumption of POP contaminated seafood (which is common among both the Swedish fishermen's families and the Inuits) may be outweighed by the positive effects of other constituents in this type of food, such as antioxidants and polyunsaturated fatty acids [39,40]. Oxidative stress in the genital tract may play a pivotal role for impaired reproductive function [41]. Excess free radical generation in spermatozoa is related to loss of motility and fertilizing potential because of peroxidation of unsaturated fatty acids in the sperm plasma membrane [41]. Furthermore, low sperm production and poor semen quality are consistent features of Se-deficient animals [42]. A higher dietary supply of antioxidants as selenium, a range of anti-oxidative vitamins as well as polyunsaturated fatty acids through seafood may outweigh deleterious effects of POPs and other toxicants that also are conveyed through seafood.

However, methodological considerations offer alternative explanations for the regional variation of fecundability and possibly some of the minor differences that were seen in semen quality. The number of contraceptive failures was much higher in the Kharkiv population. Since the TTP is not defined for unplanned pregnancies, the most fertile couples in these populations are excluded from the analyses. Thus, it seems likely that the low fecundability in the Kharkiv cohort is biased because of selection of less fertile couples. The sensitivity analysis that also included couples with contraceptive failures supported this interpretation since differences between regions became less pronounced.

The slightly higher fecundability in the Swedish sample may be explained by selection working in the opposite direction. The TTP analyses of the Swedish sample was based upon the latest planned pregnancy and thus includes the majority of assumed highly fertile couples that at other times experienced contraceptive failures – a subgroup of some 20% (Table 2). While a number of sensitivity analyses in general corroborated the reported findings, restriction to first pregnancies did reveal a reduced fecundability among the Swedish couples. Thus the observed differences in fecundability may be artefacts of design and analysis rather than effects of POP exposure or other dietary ingredients.

Time to pregnancy studies of time trends and regional differences are probably exceptionally susceptible to bias that may be difficult to identify [22,43]. Although the recall of the time taken to conceive is expected to be highly valid in the pregnancy based studies, across region differences in the average weeks (23 to 33) the women were pregnant at enrolment could cause biased comparisons of TTP if pregnancies that were terminated by a spontaneous abortion were not included on equal terms in the four regions. Earlier studies indicate links between subfecundity and spontaneous abortion [44]. Nevertheless, we believe this is a minor problem in this study because more than 86% of women were enrolled after the 12^th ^week of pregnancy and thus is at low risk for abortion. However, if exposures in addition to subfertility first of all causes early abortions out study would not pick up such effects.

The lowest sperm concentration was found in the Swedish fishermen population with high CB-153 exposure but low DDE exposure while Inuit men, high in both CB-153 and DDE, had sperm counts close to men from Warsaw and Kharkiv – both regions low in CB-153 but high or very high in DDE. The apparent low sperm count among fishermen could reflect a considerably higher age among the fishermen. Age was negatively but not statistically significant related to sperm count (p = 0.29), but inclusion of age in the model did maximally change the estimate 7 %. A more likely explanation is that the Swedish sample includes more subfertile men than the other samples where spouses of currently pregnant women were recruited. Although we only included fishermen that previously had fathered a child, the most subfertile men taking several years to achieve a pregnancy is expected to be included to a higher extent among the elderly Swedish fishermen than among the rather young men from the other study groups [25,45].

The period of sexual abstinence was lowest in Inuit men. When adjusting for abstinence the Inuits' sperm concentration and count were the highest – indicating that high levels of CB-153 and DDE are not associated with severely impaired spermatogenesis.

While the present study provides little evidence that populations with high PCB and/or DDT exposure experiences reduced sperm counts or more morphologically abnormal sperms, there are indications that sperm motility could be affected. The adjusted sperm motility was higher in the population from Warsaw with low serum level of CB-153 and moderate levels of DDE. The manual assessment of sperm motility has often been demonstrated to be highly variable [46] and bias due to differences between the four technicians that performed manual motility counting at the four study sites is a concern. However, quality control workshops performed during the study revealed an inter-individual variation in motility assessment of only 11 % among the four technicians without any indication of systematic difference between the Polish technician and the others. Thus, we do not believe that difference in laboratory methods explain the findings. Moreover, findings in this study are consistent with two independent studies based upon exposure contrasts within the Swedish population [21,23]. We also performed computer-assisted motility counting in three of the four countries (including Poland) but, unfortunately, the variability between countries in performing the analysis was too large to allow for between region comparisons.

The percentage of sperms with normal morphology did not differ significantly between countries. All morphological smears were sent to one central laboratory for staining and assessment of sperm abnormalities by two technicians. Therefore, the systematic measurement error should be minimal on this outcome, although also this outcome is known to have a high degree of intra- and inter-observer variability [46].

Cross sectional studies of semen quality suffer most often of low participation rates that may bias the internal validity of a study. In occupational semen studies, subfertility seems to be a stronger motivation for participation among referents than among exposed workers, who may have an interest to have potential harmful exposures documented. Such a differential selection may, however, be less important in environmental studies where participants may know little or nothing about their individual exposure level. In this study, the fecundability was – as expected – lower among those men that provided a semen sample than those that did not, but differences were small and – more importantly – selection was not related to levels of exposure as indicated by the study group. Moreover, the distribution of lifestyle factors, occupational exposure, and reproductive disease history among men providing semen sample and non-providers demonstrated only small differences within countries. Thus, low participation rates are not expected to bias the relation between POP exposure and semen characteristics. In Sweden, only men who agreed to donate a semen sample were interviewed making us unable to draw the same conclusions for this population. As far as the female characteristics are concerned, almost all of the listed factors differed between the four countries, but only minor differences were seen between those women whose men provided a semen sample and all other women.

Although all studies were set-up and carried out according to an agreed uniform research protocol, the marked across region differences in demographic factors as age, lifestyle factors as smoking, urogenital infections, contraceptive methods, reproductive behaviour, periods of sexual abstinence and season of sample collection call for an adequate control for potential confounding by these factors in the between regions analyses. We choose the change-in-estimate method to keep factors in the models regardless of p-values (38) and known strong determinants as age and period of abstinence were compulsory in all models. Even some residual confounding cannot be ruled out, the main threat to the internal validity of this study is probably strong selective forces that cannot be adjusted for in the analyses.

## Conclusion

In this large epidemiological study of fecundability and semen quality in regions spanning more than 10 fold differences in median serum concentrations of CB-153 and DDE, we observed regional variations in couple fecundability that are compatible with effects of DDE, but the regional variation can also be due to differences between regions with respect to recruitment, reproductive disorders, sexual behavior and use of contraception. We found no evidence that men living in areas with high POP exposure levels have low sperm counts or more abnormal sperm but men living in an area with rather low POP exposure had higher sperm motility. Subsequent work investigating the relation between individual measures of exposure and a range of fertility outcomes will hopefully provide additional insight. In addition to TTP and semen quality these studies will also include indicators of sperm chromatin structure, DNA damage and apoptosis, sexual hormones, X/Y chromosome ratio in sperm and epididymal and accessory gland function. Fertility-related selection to the semen studies with rather low participation rates seems not to be a major concern for the interpretation of our findings.

## Abbreviations

ANOVA Analysis Of Variance

CB-153 2,2',4,4',5,5'-hexachlorobiphenyl

CI Confidence Interval

CV Coefficient of variation

DDE 1,1-dichloro-2,2-bis (p-chlorophenyl)-ethylene

DDT Dichlorodiphenyl Trichloroethane

FR Fecundability Ratio

GLM General linear model

PCB Polychlorinated biphenyl

POP Persistent Organochlorine Pollutant

TTP Time to Pregnancy

WHO World Health Organisation

## Competing interests

The author(s) declare that they have no competing interests.

## Authors' contributions

JPB, AG and LH conceived and designed the project. JPB was main responsible for raising funding for the project. JPB and GT coordinated the execution of the project. ARH, GT, HSP, JKL, and VZ have been responsible for collecting all blood samples and for obtaining the interview data. GT had main responsibility for creating the joint database. GT and JPB performed the statistical analyses. GT and JPB drafted the manuscript. All authors participated in the design of the study, commented on the draft, and have read and approved the final manuscript.
